# Improving spatial-simultaneous working memory in Down syndrome: effect of a training program led by parents instead of an expert

**DOI:** 10.3389/fpsyg.2015.01265

**Published:** 2015-08-24

**Authors:** Francesca Pulina, Barbara Carretti, Silvia Lanfranchi, Irene C. Mammarella

**Affiliations:** ^1^Department of Developmental Psychology and Socialization, University of PadovaPadova, Italy; ^2^Department of General Psychology, University of PadovaPadova, Italy

**Keywords:** Down syndrome, visuospatial working memory, computer-based training, intellectual disability, memory improvement

## Abstract

Recent studies have suggested that the visuospatial component of working memory (WM) is selectively impaired in individuals with Down syndrome (DS), the deficit relating specifically to the spatial-simultaneous component, which is involved when stimuli are presented simultaneously. The present study aimed to analyze the effects of a computer-based program for training the spatial-simultaneous component of WM in terms of: specific effects (on spatial-simultaneous WM tasks); near and far transfer effects (on spatial-sequential and visuospatial abilities, and everyday memory tasks); and maintenance effects (1 month after the training). A comparison was drawn between the results obtained when the training was led by parents at home as opposed to an expert in psychology. Thirty-nine children and adolescents with DS were allocated to one of two groups: the training was administered by an expert in one, and by appropriately instructed parents in the other. The training was administered individually twice a week for a month, in eight sessions lasting approximately 30 min each. Our participants’ performance improved after the training, and these results were maintained a month later in both groups. Overall, our findings suggest that spatial-simultaneous WM performance can be improved, obtaining specific and transfer gains; above all, it seems that, with adequate support, parents could effectively administer a WM training to their child.

## Introduction

Down syndrome (DS), or trisomy 21, is the most common cause of intellectual disability of genetic origin, affecting about 1 in 700–1000 live births (e.g., McGrowther and Marshall, 1990; Sherman et al., 2007). The vast majority of individuals with DS have some degree of intellectual impairment. Despite a marked variability in terms of the severity of specific impairments (Dykens et al., 2000; Silverman, 2007), individuals with DS essentially have a profile featuring particular strengths and weaknesses. Their cognitive functioning is characterized by speech and language impairments (Chapman and Hesketh, 2000), and they often have more difficulty with expressive language than with language comprehension. Their non-verbal skills are usually less severely impaired, although recent studies have shown a variable picture that depends on which aspect of visuospatial cognition is considered (Yang et al., 2014). Several researchers have focused on working memory (WM) because of its crucial role in many everyday situations, such as learning, orientation, reasoning, and comprehension (Baddeley, 1986).

On the basis of Baddeley and Hitch’s (1974) and Baddeley’s (1986) multicomponent model, WM can be seen as a system comprising several different components. The central executive is seen as an attention-controlling system responsible for managing resources and monitoring information processing. Two slave systems are responsible for the storage of information, i.e., the phonological loop and the visuospatial sketchpad. The former is for temporarily storing and rehearsing speech-based verbal information, while the latter is for storing visuospatial information for brief periods of time.

Some researchers have analyzed WM functioning in DS, reporting impairments in executive processing (e.g., Lanfranchi et al., 2010) and the verbal component (e.g., Hulme and Mackenzie, 1992; Jarrold and Baddeley, 1997; Kittler et al., 2004), while findings on visuospatial WM are inconsistent. In fact, previous studies found participants with DS less impaired in visuospatial than in verbal WM, but more recent results suggest that individuals with DS may have difficulties in the visuospatial domain too (see Yang et al., 2014, for a review), depending on which specific aspect of this ability is considered. For instance, Lanfranchi et al. (2009) found that participants with DS performed worse than controls (children matched for mental age) in spatial-simultaneous WM tasks, but not in spatial-sequential ones. This finding can be explained in the light of the hypothesis advanced by Pazzaglia and Cornoldi (1999; see also Cornoldi and Vecchi, 2003; Mammarella et al., 2008) that sees the visuospatial sketchpad divided into three components: a visual component, involved in the recall of an object’s features; a spatial-sequential component, implicated in memory for sequentially presented information; and a spatial-simultaneous component, responsible for recalling configurations that describe simultaneously presented spatial locations.

Using this distinction, Lanfranchi et al. (2009) observed a specific deficit in DS individuals’ spatial-simultaneous WM, irrespective of the level of control required (see Cornoldi and Vecchi, 2003^1^). This result was supported by later research conducted to explore impairments in spatial-simultaneous WM more closely. Carretti and Lanfranchi (2010) found, for example, that when children with DS aged from 5 to 12 years performed spatial-simultaneous WM tasks, they did not take advantage of structured materials (in which the positions to remember formed a pattern) as effectively as a control group with typically developing (TD) children matched for mental age. To see if the DS individuals had a general problem with using structured material to memorize information, or if this problem related specifically to spatial-simultaneous WM tasks, Carretti et al. (2013) subsequently compared the advantage associated with the use of structured material in both spatial-simultaneous and spatial-sequential tasks in individuals with DS matched for mental age with TD children. Their results showed a marked difference between the two groups in the former but not in the latter tasks, confirming specific impairments in spatial-simultaneous WM in DS.

In the light of the above-mentioned findings on the particular weakness in spatial-simultaneous WM identified in individuals with DS, the aim of the present study was to investigate the feasibility of improving visuospatial WM in children and adolescents with DS. Previous studies investigating the efficacy of WM training programs in individuals with DS focused on the verbal component of WM. For example, some authors found improvements in auditory and/or visual span measures after using rehearsal training (e.g., Broadley and MacDonald, 1993; Comblain, 1994; Laws et al., 1996; Conners et al., 2001, 2008). These improvements were often limited to the skills directly treated, however. As for visuospatial WM, Bennett et al. (2013) assessed the effectiveness of a computer-based training program in reducing the memory difficulties of children with DS aged between 7 and 12 years. After approximately 3 months of training with the preschool version of the Cogmed Working Memory Training (which includes different visuospatial memory training tasks), the authors found improvements in both trained and untrained short-term visuospatial memory tasks, with no transfer to short-term verbal memory or WM skills.

In the present study, the feasibility of enhancing spatial-simultaneous WM in individuals with DS was tested using a training administered either by an expert in psychology or by parents at home. Earlier research had already demonstrated the efficacy of the training program adopted in terms of its specific effects on spatial-simultaneous WM, transfer effects on spatial-sequential WM, and maintenance effects after 1 month (Lanfranchi et al., 2014). In the present work, we focus on the person conducting the training activities because one of the problems of training administered by an expert concerns the burden on the families having to bring their child to a specialized center. With a view to the training’s applicability, it therefore seemed worthwhile to see whether giving parents guidance on how to train specific aspects of cognition (such as WM) could produce similar results to those achievable by an expert.

The training activities were conducted using a computer, partly for its motivational value, and also because previous studies had shown that it can be used effectively with DS children (e.g., Ortega-Tudela and Gómez-Ariza, 2006; Bennett et al., 2013). The final version of the training (based on Mammarella et al., 2010) consisted of activities in which memory load and attentional control were manipulated. The activities were structured to suit the cognitive profile of DS. For instance, the training involved: little verbal information and only very simple verbal instructions because DS is known to be associated with impaired verbal abilities (e.g., Rondal, 1996); practical activities because of their difficulties with abstract reasoning (e.g., Rowe et al., 2006); or simple images because they have trouble with perceptual analysis (e.g., Bellugi et al., 2000), and may have visual impairments (e.g., Dykens et al., 2000).

The present study therefore investigated whether WM training completed under the supervision of a parent could have positive effects. Conners et al. (2008) found that rehearsal training administered at home by parents was effective in improving memory span in children with DS. Although parent-implemented intervention may entail intervening variables, we agree with Conners et al. (2008) that training provided by parents can have a greater ecological validity and, if successful, improvements could be maintained by means of regular maintenance exercises.

## Materials and Methods

### Participants

Thirty-nine children and adolescents with DS (16 males and 23 females) took part in the study. Their mean chronological age was 12 years and 5 months (SD: 3 years; range: 7 years and 8 months to 19 years and 1 month). Participants were recruited from several regions in Italy through associations of parents who have children with DS, schools, pediatricians, or rehabilitation centers for people with intellectual disabilities. All participants were enrolled in mainstream school with the support of an assistant teacher. Selection criteria were: age; no severe behavioral problems; a minimum of expressive vocabulary; and the skills needed to complete the baseline assessment.

Parents’ informed written consent was obtained for all children and adolescents participating in the study.

Participants were allocated to one of two conditions: in one (Group 1 – Expert) the training was administered by an expert in psychology; in the other (Group 2 – Parent), parents were given instruction on how to administer the training at home, and they were supervised when they did so. The training activities were the same for the two groups, which were matched on a measure of non-verbal ability – Raven’s Colored Progressive Matrices (CPM; Raven et al., 1998), and on a measure of verbal ability – the Peabody Picture Vocabulary Test-Revised (PPVT-R; Dunn and Dunn, 1997). As shown in **Table 1**, non-verbal abilities are greater than verbal ones in both groups, as typically seen in individuals with DS.

**Table 1:** Participants’ characteristics.

	Group 1		Group 2		*P*	$\eta_{p}^{2}$
		
	*M*	SD	*M*	SD		
Chronological age	146.20	36.11	151.05	37.64	0.68	0.005
Colored Progressive Matrices (CPM) raw score	16.35	4.78	16.42	3.92	0.96	0.000
CPM mental age	79.05	14.35	79.26	11.76	0.96	0.000
Peabody Picture Vocabulary Test-Revised (PPVT-R) raw score	65.80	18.36	65.37	21.09	0.95	0.000
PPVT-R mental age	69.50	18.16	69.89	23.16	0.95	0.000

### Materials

To obtain measures of specific, near and far transfer, and maintenance effects, the following tasks were administered to participants at pre-test, post-test and follow-up (after 1 month).

#### Specific Effects: Spatial-Simultaneous Working Memory Tasks (Lanfranchi et al., 2004)

Each child was administered two simultaneous WM tasks, one passive and the other active.

##### Passive spatial-simultaneous task

Participants were shown for 8 s a 2 × 2, 3 × 3, or 4 × 4 square matrix where two or three squares were colored green. Immediately after the matrix was removed, they were asked to recall the positions of the green squares by pointing to the same positions on a blank matrix. This task had four levels of difficulty depending on the number of squares to be remembered (two or three) and the size of the matrix, 2 × 2 on the first level (with two green squares), 3 × 3 on the second and third (with two and three green squares, respectively), and 4 × 4 on the fourth (with two green squares). Two trials were run for each level of difficulty. A score of 1 was given for every pattern of positions recalled correctly. The final score was the sum of the scores obtained (minimum score = 0; maximum score = 8).

##### Active spatial-simultaneous task

Participants were shown for 8 s a 2 × 2, 3 × 3, or 4 × 4 matrix containing two or three red squares, and some boards also contained a blue square. Participants were then asked to remember the positions of the red squares, pointing to their locations on a blank matrix. They also had to tap on the table when a matrix containing a blue square was presented. The task had four levels of complexity, depending on the number of red squares to be remembered (two or three) and the size of the matrix: 2 × 2 on the first level (with two red squares), 3 × 3 on the second and third (with two and three red squares, respectively), and 4 × 4 on the fourth (with three red squares). Two trials were run for each level of difficulty. A score of 1 was given for every trial performed correctly, i.e., when the child remembered the position of the red squares and tapped on the table, where applicable. The final score was the sum of the scores obtained (minimum score = 0; maximum score = 8).

All the tasks were administered with a self-terminating procedure, i.e., when the child failed both trials on the same level of difficulty, the task was abandoned to avoid making participants frustrated.

#### Near Transfer Effects: Passive Spatial-Sequential Task (Lanfranchi et al., 2004)

Participants were asked to recall a path taken by a small frog on a 3 × 3 or 4 × 4 matrix, immediately after they had seen it. This task was presented with four levels of difficulty, depending on the number of steps along the frog’s path and the size of the matrix (3 × 3 for the first level with two steps, and 4 × 4 for the second, third, and fourth levels, with two, three, and four steps, respectively). The frog’s steps were presented at approximately 2-s intervals. Two trials were run for each level of difficulty. A score of 1 was given for every path recalled correctly. The final score was the sum of the scores for each trial (minimum score = 0; maximum score = 8).

#### Far Transfer Effects: Visuospatial Abilities (Geometric Puzzles) and Everyday Life (Everyday Memory Questionnaire)

##### Geometric puzzles (subtest of NEPSY battery; Korkman et al., 2007)

In this task, participants were shown a picture with a large grid containing six shapes, plus two shapes outside the grid. For each trial, the children were asked to match two shapes outside the grid with two shapes inside the grid; and they were allowed 45 s to do so. The task included 12 trials, and for each trial a score of 2 was awarded if the child correctly matched two shapes inside the grid with two outside the grid within 45 s. A score of 1 was given if a child correctly matched only one shape. The minimum score was 0 and the maximum score was 24.

##### Everyday memory questionnaire (adapted from Cornoldi et al., 2003)

Parents were asked to answer 16 questions about their child’s functioning in everyday life (e.g., Can your child remember short songs or rhymes? When he/she looks at a picture, is he/she able to remember the details?). Each item was scored using a four-point Likert-type scale (1: never or almost never, 2: sometimes, 3: often, 4: always or almost always).

### Procedure

Before parents began to administer the WM training, they met as a group and individually with a program coordinator who gave them instructions on how to conduct the training program, and explained how they should work with their child. The coordinator of this part of the project demonstrated the procedure and talked with parents about how to use strategies to sustain their children’s motivation. Parents were shown videos or PowerPoint presentations to facilitate their understanding, and they were advised to work with their child in a quiet room to minimize distractions. Parents were supervised weekly throughout the study by means of telephone calls to answer any queries about the tasks and monitor the progress of the training program. During these phone calls, the program coordinator gave parents feedback about how the activities had been carried out. If they met with any problems, parents could also contact the coordinator at any time. Finally, to check whether parents had administered the training to their child correctly, after completing the training sessions parents gave the coordinator a file with records of the training activities conducted and any progress their child had made, in terms of the activities completed correctly.

The present study was approved by the Ethical Committee of the School of Psychology at Padova University.

An ABA design was used to judge the efficacy of the training, and a follow-up assessment was performed 1 month after the post-test to identify any maintenance effects. Children in both groups first completed a pre-test assessment, when the tasks were administered over the course of 2 days during the same week. All participants started the training program within one week after the pre-test session. The training lasted 4 weeks, with two sessions a week, each session lasting about 30 min.

All participants attended a post-test session within a week after completing the training and, a month later, a follow-up assessment was conducted to check whether any improvements recorded after completing the training program had been maintained.

### Description of the Training Activities

As mentioned earlier, a computer-based training program was preferred because it seems to be effective for individuals with DS (e.g., Ortega-Tudela and Gómez-Ariza, 2006). The starting point was a training program for TD children designed to improve their visuospatial WM (Mammarella et al., 2010). This software considers two aspects of visuospatial WM: the nature of the stimulus (visual, spatial-sequential, and spatial-simultaneous; only spatial-simultaneous tasks were used in the present study); and the level of attentional control, with tasks demanding a low, medium or high level of control. Individuals with DS are weak in spatial-simultaneous WM, so our training focused exclusively on activities engaging this area. Moreover the activities were selected and adapted to the DS cognitive profile.

In particular, given these individuals’ deficit in verbal abilities (e.g., Rondal, 1996), the tasks contained little verbal information and we used very simple verbal instructions (and parents administering the training were advised to do likewise). We also used simple, concrete tasks because of DS individuals’ deficit in abstract reasoning (e.g., Rowe et al., 2006), and performing complex activities (e.g., Lanfranchi et al., 2010).

The training sessions focused alternatively on immediate attention and memory (recognition tasks), recollection (passive tasks), and active memory (active tasks that involve maintaining and processing information). In the immediate attention and memory sessions, the tasks mainly involved recognizing target stimuli; in the recollection sessions, the tasks were more complex than in the recognition tasks, and involved retrieving previously presented locations from memory; in the active memory sessions, participants were asked not only to analyze the spatial-simultaneous target stimuli, but also to maintain and process spatial-simultaneous information.

In all, there were 16 different activities. Each training session lasted approximately 30 min, and was administered twice a week. The activities proposed during the training sessions were identical for each participant in both groups.

## Results

A preliminary analysis – one-way ANOVA – revealed no significant differences between the two groups at the pre-test session for any of the measures considered (all *p* > 0.05). **Table 2** shows descriptive statistics for the measures administered.

**Table 2:** Outcome measures at pre-test, post-test, and follow-up for both groups.

	Group	Pre-test		Post-test		Follow-up	
		*M*	SD	*M*	SD	*M*	SD
Passive spatial-simultaneous working memory (WM) task	Expert	3.10	1.71	5.65	2.08	5.85	1.95
	Parent	3.89	2.33	4.68	2.29	5.37	2.11
Active spatial-simultaneous WM task	Expert	2.40	2.11	4.70	2.00	4.85	1.69
	Parent	2.79	2.32	4.16	2.41	5.32	2.06
Passive spatial-sequential task	Expert	5.55	1.93	6.40	1.43	6.50	1.57
	Parent	5.11	1.76	5.63	1.61	5.95	1.58
Geometric puzzle	Expert	14.25	5.87	16.65	5.17	16.50	5.18
	Parent	13.00	3.59	15.74	3.56	16.63	3.76
Everyday memory questionnaire	Expert	47.90	6.03	50.25	6.33	50.95	5.71
	Parent	46.84	5.69	45.79	6.31	47.21	6.09

A 3 × 2 repeated measures ANOVA, with Session (pre-test, post-test, and follow-up) as within-group factors and Group (expert- and parent-delivered training) as the between-group variables, was run on the raw scores obtained on each measure to identify specific (on spatial-simultaneous WM), near (on spatial-sequential WM) and far (on visuospatial abilities and everyday memory) transfer, and maintenance effects^2^. Interactions were analyzed using *post hoc* analyses, applying Bonferroni’s adjustment for multiple comparisons. The α-value was set at 0.05 for all statistical tests and at 0.004^3^ for interactions.

### Specific Effects. Spatial-Simultaneous Working Memory Tasks

#### Passive Spatial-Simultaneous Working Memory Task

The main effect of Session was significant (*F*_2,74_ = 57.74, *p* < 0.001, $\eta_{p}^{2}$ = 0.609), while the main effect of Group was not (*F*_1,37_= 0.12, *p* = 0.73). Participants’ performance improved from the pre-test to the post-test and follow-up sessions (*MDiff*. = –1.67, *p* < 0.001; *MDiff*. = –2.11, *p* < 0.001, respectively), while the latter two did not differ.

The Session × Group interaction was also significant (*F*_2,74_= 9.62, *p* < 0.001, $\eta_{p}^{2}$ = 0.206). Subsequent *post hoc* comparisons showed that participants in the “Expert” group performed significantly better in the post-test and follow-up sessions than in the pre-test session (*MDiff*. = –2.55, *p* < 0.001; *MDiff*. = –2.75, *p* < 0.001, respectively), with no significant differences between post-test and follow-up, indicating a maintenance effect. The “Parent” group showed significant improvements only from pre-test to follow-up (*MDiff*. = –1.47, *p* < 0.001). The two groups’ performance did not differ at pre-test, post-test, or follow-up.

#### Active Spatial-Simultaneous Working Memory Task

The main effect of Session was significant (*F*_2,74_= 61.16, *p* < 0.001, $\eta_{p}^{2}$ = 0.623). Generally speaking, participants’ performance improved from the pre-test to the post-test and follow-up sessions (*MDiff*. = -1.83, *p* < 0.001; *MDiff*. = -2.49, *p* < 0.001), but did not improve significantly from the post-test to the follow-up session. Neither the effect of Group (*F*_1,37_= 0.28, *p* = 0.867, $\eta_{p}^{2}$ = 0.001) nor the Session × Group interaction (*F*_2,74_= 2.89, *p* = 0.062, $\eta_{p}^{2}$ = 0.073) were significant.

### Near Transfer Effect

#### Passive Spatial-Sequential Working Memory Task

The main effect of Session was significant (*F*_2,74_= 14.35, *p* < 0.001, $\eta_{p}^{2}$ = 0.279). Participants’ performance generally improved from pre-test to post-test, and from pre-test to follow-up (*MDiff*. = -0.69, *p* = 0.002; *MDiff*. = -0.89, *p* < 0.001; respectively), with no significant differences between post-test and follow-up. The effect of Group was not significant (*F*_1,37_= 1.44, *p* = 0.237, $\eta_{p}^{2}$ = 0.038), nor was the Session × Group interaction (*F*_2,74_= 0.44, *p* = 0.644, $\eta_{p}^{2}$ = 0.012).

### Far Transfer Effect

#### Visuospatial Abilities (Geometric Puzzles)

The main effect of Session was significant (*F*_2,74_= 51.23, *p* < 0.001, $\eta_{p}^{2}$ = 0.581). Participants’ performance improved from the pre-test to the post-test and follow-up sessions (*MDiff*. = -2.57, *p* < 0.001; *MDiff*. = -2.94, *p* < 0.001; respectively), which did not differ. The effect of Group (*F*_1,37_= 0.22, *p* = 0.641, $\eta_{p}^{2}$ = 0.006) and the Session × Group interaction (*F*_2,74_= 2.59, *p* = 0.082, $\eta_{p}^{2}$ = 0.065) were not significant.

#### Everyday Memory Questionnaire

The main effect of Session was significant (*F*_2,74_= 4.70, *p* = 0.012, $\eta_{p}^{2}$ = 0.113). No main effects of Group emerged (*F*_1,37_= 2.88, *p* = 0.098, $\eta_{p}^{2}$ = 0.072). Participants’ performance generally improved from pre-test to follow-up (*MDiff*. = -1.71, *p* = 0.004), with no significant differences between the pre-test and the post-test sessions, or between the post-test and follow-up. The Session × Group interaction was significant, however (*F*_2,74_= 5.07, *p* = 0.009, $\eta_{p}^{2}$ = 0.121). Subsequent *post hoc* comparisons showed that the participants in the “Expert” group performed significantly better at the follow-up session than at the pre-test session (*MDiff*. = -3.05, *p* < 0.001), but there were no significant differences between the pre-test and post-test sessions (*p* = 0.022), or between the post-test and follow-up sessions (*p* = 1.00). The participants in the “Parent” group showed no significant improvement, neither from pre-test to post-test, nor from pre-test to follow-up.

**FIGURE 1:**
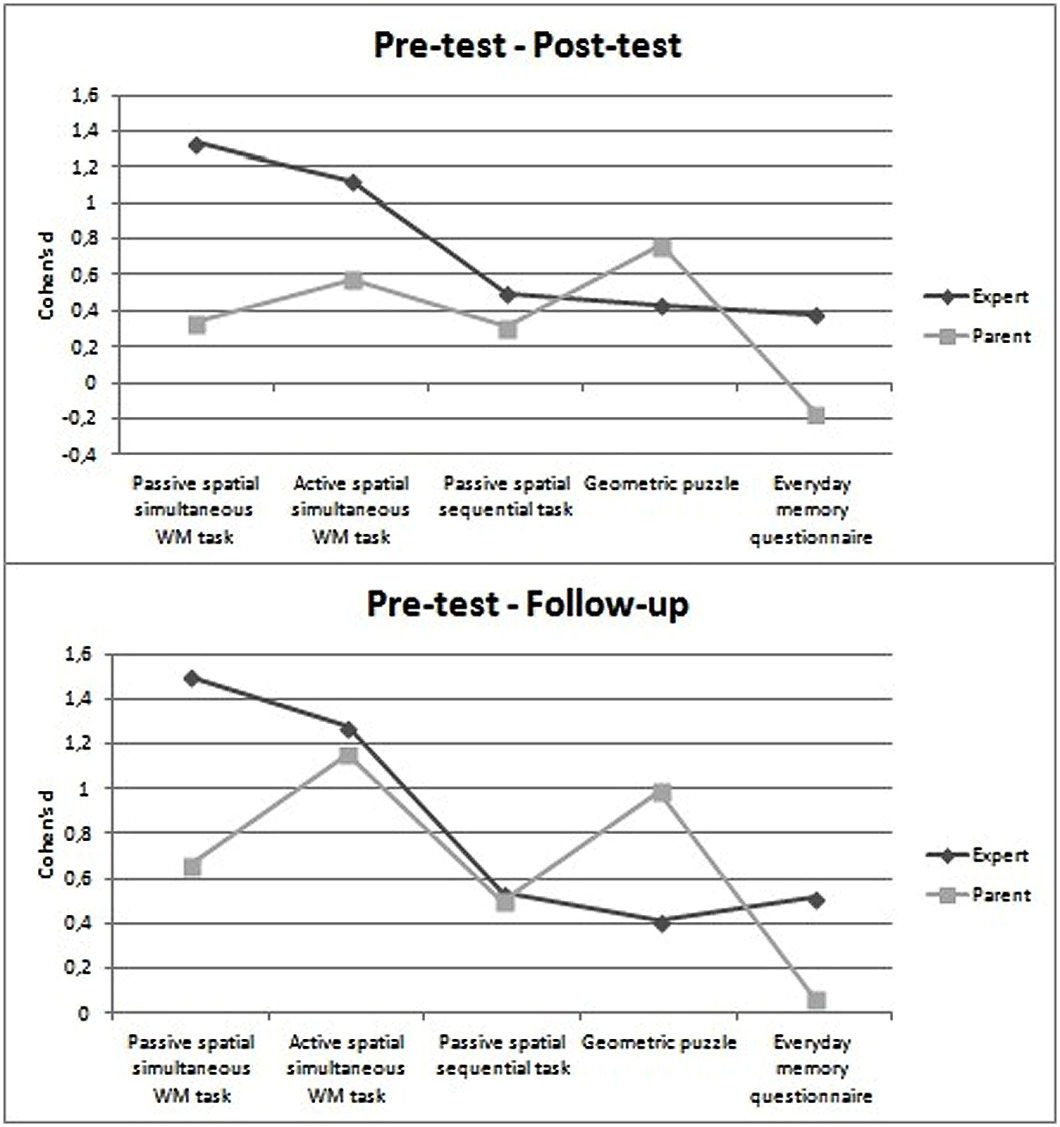
**Comparison between pre-test and post-test sessions (Upper), and between pre-test and follow-up sessions (Lower) by group, using Cohen’s *d***.

Cohen’s (1988) *d*-values were calculated to analyze the effect size of improvements from the pre-test to the post-test and follow-up sessions within each group. **Figure 1** shows the *d*-values obtained for the specific and transfer effects.

In the comparison between pre- and post-test results in the Expert group, the effect sizes were large for the passive and active spatial-simultaneous WM tasks, while they were medium for the passive spatial-sequential task, and small for the geometric puzzles and everyday memory questionnaire. In the comparison between pre-test and follow-up, the same pattern of effect sizes was apparent, with the exception of the everyday memory questionnaire for which a medium effect size was found.

In the Parent group the comparison between pre- and post-test sessions yielded medium effect sizes for the tasks testing visuospatial abilities (geometric puzzles), and active spatial-simultaneous WM, while small effect sizes emerged for the passive spatial-simultaneous and spatial-sequential WM tasks. In the comparison between pre-test and follow-up sessions, large effect sizes were found for the active spatial-simultaneous WM tasks and geometric puzzles, and medium effect sizes for the passive spatial-simultaneous and spatial-sequential WM tasks.

## Discussion

The main goal of the present study was to analyze the feasibility of improving spatial-simultaneous WM in individuals with DS by means of a computer-based training administered by parents at home. As mentioned previously, we had already tested the efficacy of the training program adopted in a previous study (Lanfranchi et al., 2014). Here, specific effects on spatial-simultaneous tasks, near transfer effects on spatial-sequential tasks, and far transfer effects on visuospatial abilities and everyday life were tested immediately after completing the training and again at a follow-up session a month later.

Judging from our results, the performance of both groups (i.e., individuals with DS trained by an expert psychologist or by their parents) improved after the training in both spatial-simultaneous WM tasks. In both cases their improvement was greater than the one seen in a passive control group in a previous preliminary study on the efficacy of our training program (Cohen’s *d* was 0.16 for the passive spatial-simultaneous task, and -0.05 for the active spatial-simultaneous task; Lanfranchi et al., 2014). The time it took for the improvement to become apparent differed between the two groups, however.

Our most important finding lies in that parents were able to administer the training to their children, who benefited from the intervention: participants in the “Parent” group showed significant improvements in performing the passive spatial-simultaneous task. This was only true, however, for the comparison between the pre-test and the follow-up, whereas no improvement emerged immediately after completing the training. In contrast, participants in the “Expert” group performed better already at the post-test stage, and maintained this gain a month later. In other words, participants in the “Parent” group seemed to improve more gradually. The benefits of the training, in terms of specific effects, only became evident with time. The different rate of improvement in the two groups might mean that the expert was more effective in promoting changes in performance; parents would probably need more time to become familiar with the training activities. The improvement in the “Parent group” that emerged at the follow-up session might be related to changes in the way parents interacted with their children, producing “pervasive” effects on their performance that became apparent at the follow-up assessment. In other words, parents may have helped their children learn to pay more attention to details, or to use more appropriate strategies – even outside the context of the training activities.

In contrast, no differences emerged between the two groups in the active spatial-simultaneous task: both groups performed better at post-test then at pre-test, and their improvement was maintained a month after completing the training.

In addition to the specific effects, the training also induced near and far transfer effects in both groups. Concerning the near transfer effects, there was some improvement in a WM component that was not treated specifically, i.e., spatial-sequential WM. Participants performed better at the post-test than at the pre-test session and this improvement was also maintained a month later. Similar findings emerged for the geometric puzzles task. Here again, the two groups improved from the pre-test to the post-test session, and maintained their better performance after 1 month. Taken together, these results confirm that the type of training considered here could be administered by parents just as effectively as by an expert.

On the whole, the specific and transfer effects identified here can be explained in terms of strategy acquisition: during the training activities, participants were stimulated to adopt appropriate strategies to solve the tasks, and to generalize them to other tasks.

Concerning the everyday memory questionnaire, our findings differed between the two groups: the “Expert” group improved from the pre-test to the follow-up, indicating that parents’ opinions of their children’s everyday functioning became more positive after the training. In the “Parent” group, on the other hand, participants’ scores in the questionnaire showed no significant differences between the three sessions; they dropped slightly from pre-test to post-test, then returned to the same level as at the pre-test in the follow-up session. A possible explanation for these results lies in that, having received specific instruction, the parents concerned were more aware of their child’s abilities and difficulties, and were consequently more severe in the opinions they expressed. In other words, the lower scores would indicate not a worse everyday functioning of the participants, but a change in their parents’ awareness of their memory ability.

In general, our study demonstrated the feasibility of improving WM performance in children and adolescents with DS, even with a relatively short training program and when the training is administered by parents. The effects of the training were not limited to the specific area of WM treated, but were also generalized to other skills, as demonstrated by near and far transfer effects. To our knowledge, this is one of the few studies to have attempted an analysis on the effect of visuospatial WM training in DS. In a recent study, for instance, Bennett et al. (2013) tested the efficacy of a computer-based training (that involved different visuospatial memory tasks) in reducing memory difficulties in children with DS. They reported improvements in both trained and untrained short-term visuospatial memory tasks and, in some children, also in tasks measuring executive functions, as indicated by parents’ responses to the BRIEF-P (Gioia et al., 2003). In contrast, they reported finding no transfer effects on verbal short-term memory and verbal WM skills. The findings obtained by Bennett et al. (2013) and our own results reported here support the feasibility of computer-based training programs enhancing visuospatial WM in individuals with DS, and also obtaining transfer effects.

In our opinion, the results of the present study are important for several reasons. For a start, having demonstrated the effectiveness of this training even when it is administered by parents show that it could be used more frequently and/or periodically in order to maintain the effects of the training. The other point of interest concerns the confirmation of its efficacy in everyday life functioning (such as learning activities, reasoning, orientation, etc.), the training program could prove useful in clinical and rehabilitation settings.

Beyond the results obtained here, it could be interesting in future studies to analyze the nature of the effects, and particularly of the transfer effects, more systematically. For example, it would be important to examine the effects on verbal WM or other domains of cognitive functioning, especially those related to everyday life. Further research could shed more light on this issue, which is a source of debate in the literature (e.g., Melby-Lervag and Hulme, 2013).

## Conclusion

In line with the results reported by Conners et al. (2008), who found that training led by parents could produce positive effects on memory performance, our findings suggest that – with adequate support and instruction – parents of individuals with DS can administer their offspring effective WM training programs. In our research, we identified the same specific improvements in spatial-simultaneous WM, and the same transfer and maintenance effects, as when the training activities were administered by an expert psychologist.

## Conflict of Interest Statement

The authors declare that the research was conducted in the absence of any commercial or financial relationships that could be construed as a potential conflict of interest.

## Footnotes

1 The Cornoldi and Vecchi (2003) model is based on the hypothesis that both verbal and visuospatial WM tasks can be described on two continuous dimensions: a horizontal continuum that refers to the type of stimulus (verbal, visual, spatial-simultaneous, and spatial-sequential); and a vertical continuum referring to the level of control, some (active) tasks requiring a higher level of WM control than others (passive tasks).

2 All the analyses were also run with chronological age as a covariate, and the results remained substantially the same

3 For the interactions, the alpha value for *post ho*c comparisons was set at 0.004 because 12 comparisons were conducted (0.05/12 = 0.004).
